# Time to Harmonize Dengue Nomenclature and Classification

**DOI:** 10.3390/v10100569

**Published:** 2018-10-18

**Authors:** Lize Cuypers, Pieter J. K. Libin, Peter Simmonds, Ann Nowé, Jorge Muñoz-Jordán, Luiz Carlos Junior Alcantara, Anne-Mieke Vandamme, Gilberto A. Santiago, Kristof Theys

**Affiliations:** 1 Department of Microbiology and Immunology, Rega Institute for Medical Research, Clinical and Epidemiological Virology, KU Leuven, 3000 Leuven, Belgium; lize.cuypers@kuleuven.be (L.C.); pieter.libin@vub.ac.be (P.J.K.L.); annemie.vandamme@kuleuven.be (A.-M.V.); 2 Nuffield Department of Medicine, University of Oxford, Oxford OX1 3SY, UK; peter.simmonds@ndm.ox.ac.uk; 3 Artificial Intelligence Lab, Department of Computer Science, Vrije Universiteit Brussel, 1050 Brussels, Belgium; ann.nowe@ai.vub.ac.be; 4 Division of Vector-Borne Diseases, Dengue Branch, Centers for Disease Control and Prevention, San Juan, PR 00920, USA; jmunoz@cdc.gov (J.M.-J.); gsantiago@cdc.gov (G.A.S.); 5 Laboratório de Flavivírus, Instituto Oswaldo Cruz, FIOCRUZ, 21040-360 Rio de Janeiro, Brazil; luiz.alcantara@ioc.fiocruz.br; 6 Global Health and Tropical Medicine, Unidade de Microbiologia, Instituto de Higiene e Medicina Tropical, Universidade Nova de Lisboa, 1349-008 Lisbon, Portugal

**Keywords:** dengue virus, classification, nomenclature, diversity

## Abstract

Dengue virus (DENV) is estimated to cause 390 million infections per year worldwide. A quarter of these infections manifest clinically and are associated with a morbidity and mortality that put a significant burden on the affected regions. Reports of increased frequency, intensity, and extended geographical range of outbreaks highlight the virus’s ongoing global spread. Persistent transmission in endemic areas and the emergence in territories formerly devoid of transmission have shaped DENV’s current genetic diversity and divergence. This genetic layout is hierarchically organized in serotypes, genotypes, and sub-genotypic clades. While serotypes are well defined, the genotype nomenclature and classification system lack consistency, which complicates a broader analysis of their clinical and epidemiological characteristics. We identify five key challenges: (1) Currently, there is no formal definition of a DENV genotype; (2) Two different nomenclature systems are used in parallel, which causes significant confusion; (3) A standardized classification procedure is lacking so far; (4) No formal definition of sub-genotypic clades is in place; (5) There is no consensus on how to report antigenic diversity. Therefore, we believe that the time is right to re-evaluate DENV genetic diversity in an essential effort to provide harmonization across DENV studies.

## 1. Letter

Dengue virus (DENV) is hierarchically classified into serotypes (DENV-1–4) and subsequently into a large number of genotypes and sub-genotypic clades. As we examine here, inconsistencies and challenges in the DENV taxonomy are becoming increasingly apparent, which complicates investigations of virus epidemiology and pathogenicity. With this letter, we want to raise a community response to critically revise the current nomenclature and classification of DENV and to establish a widespread consensus. An effort long due, as the DENV pandemic has assumed dramatic proportions over the last 10 years [1].

Dengue virus is estimated to cause more than 300 million infections per year worldwide [2]. A quarter of these infections manifest clinically and are associated with a morbidity and mortality that put a significant burden on the affected regions [3]. Reports of increased frequency, intensity, and extended geographical range of outbreaks highlight the global importance of dengue [4]. An important contributor to this spread is the expansion of the mosquito vectors (i.e., *Aedes aegypti* and *Aedes albopictus*) into new areas. This expansion is facilitated by increased globalization [5], intensified transportation [6], the transformation of urban environments [7], and climate change [8]. Efforts by the International Committee on Taxonomy of Viruses (ICTV) to re-evaluate *Flaviviridae* taxonomy have led to well-defined DENV serotypes [9]. Within each serotype, different genotypes can be defined, which have various degrees of genetic complexity. Preceding studies have shown that DENV genotypes may migrate, establish transmission in new areas, adapt to mosquito vectors and human hosts, and alter the epidemiology and clinical outcomes of the disease [10,11,12]. Decades of persistent transmission of DENV in endemic areas, migration and displacement of genotypes, and emergence of the virus in territories formerly devoid of transmission have shaped DENV’s genetic diversity and divergence [13,14], insufficiently comprised by the available genotype nomenclature and classification systems. This lack of consistency complicates the general understanding of DENV diversity and the possible association of clinical and epidemiological characteristics associated with the genetic composition of the virus. Here we present five key challenges that underlie the current predicament with respect to DENV nomenclature and classification, a situation that has historically grown through 20 years of unaligned research efforts.

**Figure 1:**
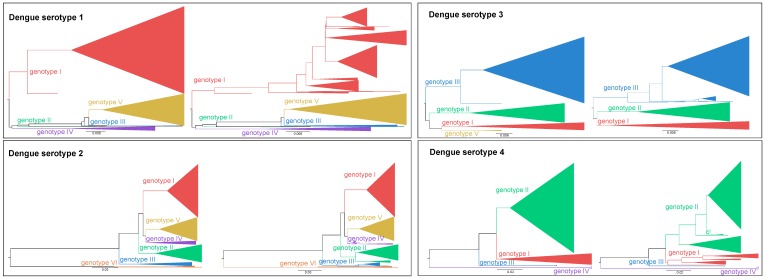
Exploration of the maximum pairwise genetic distance to define genotypes within Dengue serotypes. For each Dengue serotype, a dataset of full-length genome sequences (for more details, see Supplementary Materials) was assembled from Genbank. Multiple sequences alignments [17] were used to infer maximum-likelihood trees (see Supplementary Materials). Genotype classification was performed using an automated online phylogenetic tool [18] based on the current classification and visualized on the left side for each serotype with viral strains coloured based on genotype. On the right side, Clusterpicker [19] was used to classify viral strains in clusters based on bootstrap support and genetic distance. For all Dengue serotypes a bootstrap support threshold of 90% and a genetic distance of 7% (as previously defined [16]) were used. Scattering of currently classified genotypes shows that there is a clear need to revise the definition of DENV genotypes.

Firstly, there is currently no formal definition of a DENV genotype. Sub-serotypic taxonomic groups with monophyletic diversity (i.e., genotypes) were reported as soon as the first DENV genome sequences were generated [15]. Genotypes, for DENV-1 and DENV-2, were initially described as clades with maximum pairwise sequence distances of 6% [15]. Other DENV-2 taxonomic studies report inter-genotype and intra-genotype diversity of 7.3% and 2.6%, respectively [16], indicating the need for a re-assessment of distance-based criteria. Due to the lack of a formal definition, researchers continue to use ad hoc genotype descriptions, which results in inconsistencies with respect to the set of reported genotypes and their makeup. Exploring the pairwise genetic distance of genotypes within the four serotypes confirms the need to revise the taxonomy of sub-serotypic groups, as many genotypic clades that cover more than 7% genetic distance can be demonstrated by the scattering of predefined genotypes into several subclades (Figure 1).

Secondly, two different nomenclature systems are currently used in parallel, which causes significant confusion. One system labels genotypic clades by their geographic origin while the other uses Roman-numeric labels (e.g., the same DENV-3 genotype is referenced to as “Indian Subcontinent” or “genotype III” throughout different studies [10,20,21]). Additionally, a nomenclature system based on geographical association becomes outdated as genotypes are introduced in different geographical regions, where they continue to diverge (e.g., the DENV-3 Indian Subcontinent genotype is now endemic in the Americas [22]). As the dispersal of Dengue virus is expected to remain a global process, we propose to move towards a uniform nomenclature system based on numerical labels, proven to be appropriate for other viruses within the family of *Flaviviridae* [9].

Thirdly, a standardized classification procedure that could assign sequences to a previously defined genotypic/sub-genotypic clade is currently lacking. Genotypic classifications are generally based upon phylogenetic analysis while phylogenies inferred from whole-genome sequence alignments are considered the gold standard; in many studies the available genomic region is limited to only one or a few genes. This is particularly worrisome since significant disparities have been observed between phylogenies inferred from partial and whole-genome sequence data. To demonstrate this disparity, we performed a phylogenetic analysis on a large dataset (*n* = 3793, for details see Supplementary Materials: “Dataset and alignment” section) that highlights three major concerns with respect to sequence classification:

(a) The phylogenetic signal of the different DENV genes was assessed (see Supporting Information: Figure S1a). This signal is a measure of statistical dependence among species due to their phylogenetic relationships and is associated with the accuracy of phylogenetic studies [23]. The results of this analysis indicate that only parts of the DENV genome are suitable to classify sequences, at genotype and sub-genotype level, with high confidence. Although the envelope gene region is most often used for classification purposes, given its historical, diagnostic and functional importance, our evaluation shows that other genetic regions, such as NS1, NS3, and NS5 exhibit higher phylogenetic support. Whole-genome sequences provide superior classification precision and their availability is expected to increase in the near future when next-generation sequencing becomes routine practice, which will create an opportunity to harmonize DENV classification. Therefore, a detailed analysis of the classification potential of different genomic regions (as well as combinations of such genomic regions) is imperative to propose an adequate classification protocol.
(b) We identified that particular clades are not clustering consistently over the entire genome and established these to be clades with a potential recombinant origin (see Supplementary Materials: Figure S1b). This highlights the need for any future classification protocol to assess the recombination signal of strains by identifying recombinant breakpoints prior to their classification. In addition, it illustrates the necessity to carefully select reference strains to perform consistent and sound classifications, in contrast to the widespread ad hoc classification in much of the current literature.
(c) Certain whole-genome strains do not cluster with any known genotype. As we verified that these strains are not inter-genotypic recombinants (for details see Supplementary Materials: “Dataset and alignment” section), these strains appear to be outliers that the currently described genotypes fail to cover (see Supplementary Materials: Figure S1c and Table S1). To improve our understanding of their origin and whether these outliers indicate the source of novel genotypic or sub-genotypic clades, an in-depth analysis considering both a representative dataset and formal genotype definition is warranted.

Fourthly, no formal definition of sub-genotypic clades or lineages, either with respect to nomenclature or classification, is in place. Harmonizing sub-genotypic clades into a well-defined, systematic classification scheme will facilitate researchers to describe the increasing intra-genotypic genetic diversity, which is vital to cover the growing epidemic and its associated diversity [24,25].

Finally, there is a need to consistently report antigenic variation. Recent work shows that serotype classification insufficiently explains antigenic differences and that antigenic clusters may even transcend serotypic boundaries [26]. It is necessary to consider this variation when classifying DENV lineages, as antigenic diversity is associated with the intensity of DENV epidemics and DENV disease severity [26].

The time is right to re-evaluate DENV genetic diversity in an effort that will benefit greatly from the thousands of DENV genome sequences already available in the public domain. Advances in methodologies for DENV surveillance and guided sampling strategies, as well the potential use of DENV whole-genome sequencing in a clinical and epidemiological context, further illustrates the urgency to question the current DENV taxonomy. Here, our primary intent is to raise awareness of the need for a re-evaluation by identifying challenges that affect the increasing importance of DENV genomics in understanding virus disease manifestations and epidemic spread. A re-evaluation will provide harmonization across DENV studies and guide scientists to construct tools to detect outbreaks and infer epidemiological trends. A study group has been initiated within the structure of the ICTV (Study group page: https://talk.ictvonline.org/ictv_wikis/flaviviridae/w/sg_flavi/999/dengue-virus) to study the challenges raised in this letter in depth and to propose a new classification scheme for DENV. We welcome all scientists who would like to contribute to contact us via this forum.

## Supplementary Materials

The following materials are available online at http://www.mdpi.com/1999-4915/10/10/569/s1, Figure S1: Phylogenetic analysis that highlights three major concerns with respect to sequence classification. A phylogenetic analysis was conducted on a dataset consisting of 3793 whole-genome sequences. Firstly, an in-depth assessment of the phylogenetic signal was performed (Figure S1a). This analysis demonstrates that only parts of the DENV genome are suitable to classify sequences with high confidence. Secondly, we evaluated differences in clustering patterns among trees inferred from different genetic regions and demonstrate clades with a potential recombinant origin (Figure S1b). This highlights the need to assess the recombination signal of both the reference strains and the query strains to be classified. Thirdly, we identified outliers that do not cluster with any known genotype (Figure S1c), for which accession numbers are provided in Table S1. This indicates that only part of DENV’s genetic diversity is covered by the genotypes as they are currently described.
