# Supplementary material for: Time to Harmonize Dengue Nomenclature and Classification
Source: Viruses. 2018 Oct 18;10(10):569. doi: 10.3390/v10100569 (PMC6213058; doi:10.3390/v10100569)
Supplement: Supplementary file 1 [file viruses-10-00569-s001.docx]

Communication

Time to Harmonize Dengue Nomenclature and Classification

Lize Cuypers, Pieter J.K. Libin, Peter Simmonds, Ann Nowé, Jorge Muñoz-Jordán, Luiz Carlos Junior Alcantara, Anne-Mieke Vandamme, Gilberto A. Santiago and Kristof Theys

**Supporting information:**

1. Dataset and alignment

A dataset of whole-genome sequences, encompassing the four DENV serotypes, was collected from GenBank. After performing an in-depth quality assessment, the respective datasets contained 1555 serotype 1, 1215 serotype 2, 850 serotype 3 and 173 serotype 4 strains. Sequence alignments were constructed using an *in-house* codon-aware alignment tool [1]. We performed an initial screening for recombinant sequences using the RDP4 software [2]. We removed sequences that were identified as recombinants from the alignments.

**Figure S1: Phylogenetic analysis that highlights three major concerns with respect to sequence classification.**

1. An assessment of the phylogenetic signal of the 3793 sequences, using the likelihood-mapping algorithm implemented in TreePuzzle [3], suggests that not all sub-genomic regions are suitable for high confidence classification purposes (see Figure S1a). The strength of the phylogenetic signal is visualized as a gradient along the DENV genome, with the lighter the blue, the less phylogenetic signal is observed (see legend in Figure S1a). The percentage of phylogenetic support is required to be higher than 90% to obtain reliable conclusions deduced from phylogenetic analyses [4]. Although the envelope region is most often used for classification purposes in general research settings, our evaluation shows other genetic regions (i.e., NS1, NS3 and NS5) exhibit higher phylogenetic support (>95%).
2. Following up on the initial screening for recombination, a phylogenetic tree of the 3793 sequences was constructed, per serotype and per protein (i.e., core, membrane, envelope, and non-structural proteins NS1, NS2A, NS2B, NS3, NS4A, NS4B and NS5), to compare the topologies of the different gene fragments using the whole-genome topology as a reference. The phylogenetic trees were inferred with maximum-likelihood phylogenetic analysis under a GTR+Γ substitution model using RAxML [5]. Tree robustness was evaluated by generating 1000 bootstrap replicates. The trees were rooted using a different serotype as outgroup. This analysis revealed that particular clades cluster differently, depending on the region used. As an example, we show the comparison between the phylogenetic trees inferred from the DENV3 whole-genome and from the DENV3 NS5 region (shown in Figure S1b: upper panel). All genotypes of the DENV3 whole-genome are visualized: genotype (GT) 1 (red), GT2 (green), GT3 (blue) and GT5 (yellow). The query sequences are clustering with GT1 in the whole-genome tree, while in the phylogeny based on the NS5 region they do no longer cluster together with the red clade (GT1), but now with the green clade (GT2). (Note that, to improve the presentation clarity, some portions of the trees were collapsed.)

These observations indicate that despite the screening for putative recombinant strains using state-of-the-art methodology (i.e. RDP4), the persistent presence of low bootstrap values and an atypical tree topology indicate the existence of potential additional recombination events at the origin of some clades. This is comparable to what was shown for HIV, where recombination also confounds the origin of some clades [6]. We investigated this hypothesis using a bootscan analysis with SimPlot [7], as shown in Figure S1b (lower panel). A sliding window of 400 nucleotides with step size of 40 nucleotides was used to analyse the phylogenetic relationship between the query sequences and the other clades (GT1-5). These clades, numbered according to the NS5 tree in the upper panel of Figure S1b, are coloured in red (GT1), green (GT2), blue (GT3) and yellow (GT5).

1. Certain strains do not cluster within any genotype, demonstrating that the current genotype descriptions fail to cover the observed spectrum of DENV genetic diversity (Figure S1c). We considered five DENV1 genotypes coloured in red (genotype I), green (genotype II), blue (genotype III), purple (genotype IV) and yellow (genotype V). The strains that do not cluster with any of these genotypes (i.e. the outliers) were coloured in grey. We confirmed the strains indicated in grey not to be recombinants and more details are listed in Table S1.

For this analysis, we selected a subset of our dataset. We performed a pairwise comparison between all sequences in the dataset. Only sequences that had a genetic distance of 2% or more compared to all other sequences were included. The included sequences were used to infer a phylogenetic tree with maximum-likelihood phylogenetic analysis under a GTR+Γ substitution model using RAxML [5]. Tree robustness was evaluated by generating 1000 bootstrap replicates. Only bootstrap values higher or equal to 70% are shown on the tree.

**Figure S1a: Phylogenetic signal across the DENV-1 genome.**

**Figure S1b: Phylogenetic topologies across DENV3 genetic regions.**

Upper panel: DENV3 phylogenetic trees inferred from the whole-genome alignment (left) and NS5 protein alignment (right) are shown. Lower panel: Visualization of the Simplot analysis results with the blue clade as query strain, clustering either with genotype (GT) 1 in the whole-genome panel and with GT2 in the NS5 panel. The genotypes are marked in the following colors: GT1 in red, GT2 in green, GT3 in blue and GT5 in yellow.

**Figure S1c: DENV1 whole-genome outliers.**

The respective accession numbers of the strains here defined as outliers are listed in Table S1.

**Table S1: List of accession numbers of the sequences defined as outliers in Figure S1c.**

| Outliers | | |
| --- | --- | --- |
| AY732483 | Zhang *et al*. J Virol 2005 [8] | Report of a major clade replacement event in DENV1 genotype I |
| AF350498 | Unpublished |  |
| JQ922545 | Unpublished |  |
| KP723476 | Zhao *et al*. PLoS One 2016 [9] | Dengue outbreak in Guangdong, China – classified as genotype I |

References

1. Libin, P.; Deforche, K.; Theys, K.; Abecasis, A. VIRULIGN: Fast codon-correct alignment and annotation of viral genomes. *Bioinformatics* **2018**. DOI:10.1101/409052.
2. Martin, D.P.; Murrell, B.; Khoosal, A.; Muhire, B. Detecting and analyzing genetic recombination using RDP4. *Methods Mol Biol* **2017,** *1525*, 433-60. DOI: 10.1007/978-1-4939-6622-6.
3. Schmidt, H.A.; Strimmer, K.; Vingron, M.; von Haeseler, A. TREE-PUZZLE: maximum likelihood phylogenetic analysis using quartets and parallel computing. *Bioinformatics* **2002**, *18,* 502-4. DOI: 10.1093/bioinformatics/ 18.3.502.
4. Schmidt, H.A., Petzold, E.; Vingron, M.; von Haeseler, A. Molecular phylogenetics: parallelized parameter estimation and quartet puzzling. *J Parallel Distrib Comput* **2003**, *63*, 719-27. DOI: 10.1016/S0743-7315(03)00129-1.
5. Stamatakis, A. RAxML version 8: a tool for phylogenetic analysis and post-analysis of large phylogenies. *Bioinformatics* **2014**, *30*, 1312-3. DOI: 10.1093/bioinformatics/btu033.
6. Abecasis, A.B.; Lemey, P.; Vidal, N.; *et al*. Recombination confounds the early evolutionary history of human immunodeficiency virus type 1: subtype G is a circulating recombinant form. *J Virol* **2007**, *81*, 8543-51. DOI: 10.1128/JVI.00463-07.
7. Lole, K.S.; Bollinger, R.C.; Paranjape, R.S.; *et al*. Full-length human immunodeficiency virus type 1 genomes from subtype C-infected seroconverters in India, with evidence of intersubtype recombination. *J Virol* **1999**, *73*, 152-60.
8. Zhang, C.; Mammen, M.P. Jr.; Chinnawirotpisan, P.; *et al*. Clade replacements in dengue virus serotypes 1 and 3 are associated with changing serotype prevalence. *J Virol* **2005**, 79, 15123-30. DOI: 10.1128/JVI.79.24.15123-15130.2005.
9. Zhao, H.; Zhang, F.C.; Zhu, Q.; *et al*. Epidemiological and virological characterizations of the 2014 Dengue Outbreak in Guangzhou, China. *PLoS One* **2016**, *11*. DOI: 10.1371/journal.pone.0156548.
